# Alcohol Consumption and Oxidative DNA Damage

**DOI:** 10.3390/ijerph8072895

**Published:** 2011-07-14

**Authors:** Takeshi Hirano

**Affiliations:** Department of Life and Environment Engineering, Faculty of Environmental Engineering, The University of Kitakyushu, 1-1 Hibikino, Wakamatsu-ku, Kitakyushu, Fukuoka 808-0135, Japan; E-Mail: t-hirano@env.kitakyu-u.ac.jp; Tel.: +81-93-695-3206; Fax: +81-93-695-3299

**Keywords:** alcohol, 7,8-dihydro-8-oxoguanine, 8-oxoguanine DNA glycosylase 1, reactive oxygen species, vitamin-depleted diet

## Abstract

To examine the effects of alcohol consumption on cancer risk, we measured oxidative DNA damage and its repair activity in the livers and esophagi of rats fed with ethanol. Using our previously designed protocol for feeding rats with a high concentration of ethanol, we examined the effects of ethanol consumption on 8-oxo-Gua generation and repair activity in the livers and esophagi of rats. We found that a high concentration of ethanol accompanied with a vitamin-depleted diet increased 8-oxo-Gua and its repair activity. 8-Oxo-Gua is known to induce point mutations, leading to carcinogenesis. Therefore, these results suggested that a high concentration of ethanol and an irregular diet increased liver and esophageal cancer risk. On the other hand, we showed that a low concentration of ethanol decreased 8-oxo-Gua and its repair activity in the livers of mice treated with a carcinogen. Taken together, the effects of ethanol consumption on cancer risk depend on the ethanol concentration and the diet pattern.

## 1. Introduction

An association between chronic ethanol consumption and cancer risk has been shown by epidemiological studies. Recent studies have revealed that alcohol consumption was associated with an increase in breast cancer incidence in women [1,2], esophageal cancer [3], and colorectal cancer [4,5]. Ethanol is normally metabolized to acetaldehyde, by oxidative transfer of the hydrogen of the ethanol by alcohol dehydrogenase (ADH) to nicotinamide dinucleotide (NAD^+^), which is reduced to NADH, and by the microsomal ethanol oxidation system (MEOS). Xanthine oxidase oxidizes the acetaldehyde formed by ethanol metabolism and generates reactive oxygen species (ROS). Therefore, ethanol is capable of generating ROS during its metabolism. ROS are highly reactive, because they possess an unpaired electron. It is well known that ethanol increases the production of superoxide anions and hydroxyl radicals, which react rapidly with biological materials, causing oxidative damage in living organisms [6].

Long-term alcohol consumption is a major risk factor for liver disease in humans. The consumption of large amounts of ethanol over a long period can lead to liver cirrhosis and dysfunction. Liver dysfunction can also inhibit the detoxification of carcinogenic compounds that are ingested. By generating ROS, ethanol may affect the nutritional status, liver function, immune function, and other body functions, and may influence the risks for various types of cancer.

**Figure 1:**
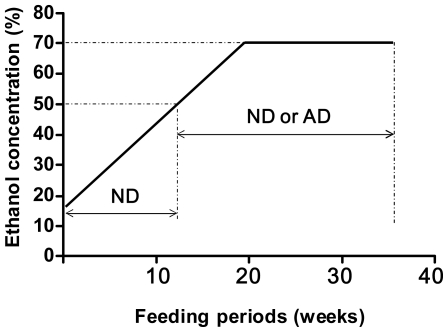
Experimental protocol for ethanol administration. The ethanol concentration was increased from 12 to 70% over a 20-week period. Once the concentration reached 50%, ND and AD were given every other week. ND: normal diet, AD: autoclaved diet.

It is well known that ROS cause damage to DNA. Among the various forms of oxidative DNA damage, 7,8-dihydro-8-oxoguanine (8-oxoguanine, 8-oxo-Gua) is a major form and a useful marker of cellular oxidative stress [7]. Studies of oxidative DNA damage should be helpful in elucidating the mechanisms of cancer induction by alcohol consumption. Previously, we designed a protocol for feeding rats with a high concentration of ethanol and a vitamin-depleted diet, to induce liver fibrosis [8]. This protocol simulated the drinking and eating patterns of heavy alcohol drinkers, who tend to eat unbalanced meals at irregular intervals. Additionally, most of the heavy drinkers had started drinking when they were less than 20 years old. Based on this information, we fed young (3-week-old) rats an initial concentration of 12% ethanol and increased the concentration up to 70% with a vitamin-depleted diet (autoclaved diet, AD) or a normal diet (ND) (Figure 1). AD was prepared by autoclaving ND for 30 min at 121 °C. The nutritional value of the diet is shown in Table 1. The reason why the ethanol concentration was increased by 3%/week is that rats need training to drink a high concentration of ethanol; otherwise, they refuse to drink 70% ethanol. By using this protocol, we observed increases in the 8-oxo-Gua levels and its repair activity in the livers of rats after long-term alcohol- and AD-feeding [6]. In addition, we found increases in the 8-oxo-Gua levels and its repair activity in the esophagi of rats given long-term ethanol and AD [9]. In this article, we will describe the association between alcohol consumption and the generation of 8-oxo-Gua, by summarizing our previous work [6,9,10].

## 2. 8-Oxoguanine

**Figure 2:**
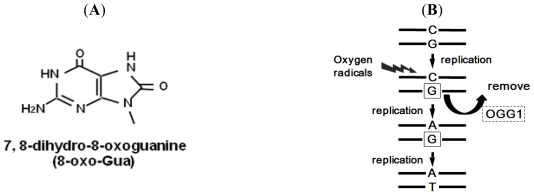
(**A**) Structure of 8-oxo-Gua. 8-Oxo-Gua is formed by the hydroxylation of guanine at the C-8 position; and (**B**) Mechanism of GC-to-TA point mutation induction. OGG1 is 8-oxoguanine DNA glycosylase, which removes 8-oxo-Gua generated in DNA. 
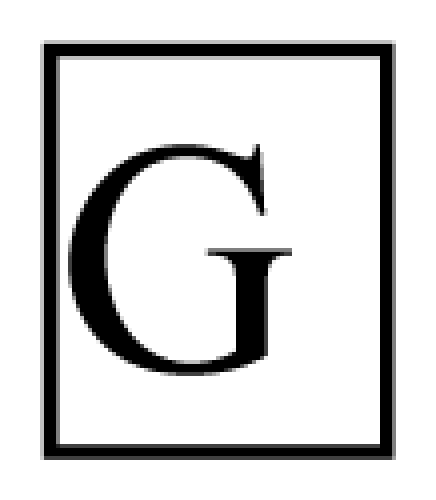
 : 8-oxo-Gua.

8-Oxo-Gua is a mutagenic lesion formed spontaneously in the genomic DNA of aerobic organisms (Figure 2A) and by the actions of exogenous factors, such as ionizing radiation, chemical pollutants, metals, food, and bacteria. Although 8-oxo-Gua is not necessarily the most abundant form of oxidative DNA damage, it has been the most extensively studied, because it can be quantitated with high sensitivity by high performance liquid chromatography coupled with electrochemical detection (HPLC-ECD), and is quite easily measured in laboratories [11,12]. We have studied the relationship between the 8-oxo-Gua levels and health-related factors, such as chemical agents [13–15], γ-irradiation [16], aging [17–19], and physical exercise [20,21]. 8-Oxo-Gua is thought to be responsible for carcinogenesis, because it induces the GC-to-TA transversion type point mutation in DNA *in vitro* [22,23]. In fact, several reports have supported this premise, based on experiments with cultured cells and with animals [13–15,24,25]. Therefore, analyses of 8-oxo-Gua generation are useful to understand the detailed mechanisms of carcinogenesis.

## 3. 8-Oxoguanine Repair System

DNA repair systems maintain the integrity of the mammalian genome by removing DNA damage, reducing the mutation frequency of cancer-related genes, minimizing replication errors, and curtailing deleterious rearrangements arising via aberrant recombination [26]. If 8-oxo-Gua is not repaired, then it leads to the accumulation of GC-to-TA point mutations (Figure 2B), suggesting that 8-oxo-Gua may play a key role in carcinogenesis [22,27–29]. From the earliest stage of 8-oxo-Gua research, repair systems for 8-oxo-Gua were predicted to exist [30].

Since 8-oxo-Gua was reported in 1984 [7], many researchers have tried to detect and clone its repair enzymes. An endonuclease nicking assay indicated the existence of a repair system for 8-oxo-Gua in a wide variety of living organisms, including bacteria, yeast, mammals, and even plants. The first information was obtained from studies of the enzyme formamidopyrimidine DNA glycosylase (Fpg or MutM), which excises 8-oxo-Gua, 2,6-diamino-4-hydroxy-5-formamidopyrimidine (FapyGua), and 4,6-diamino-5-formamidopyrimidine (FapyAde) from the DNA of *Escherichia coli* [31]. In 1996, an Fpg homologue was identified in *Saccharomyces cerevisiae* [32,33]. This was the first report for 8-oxoguanine DNA glycosylase 1 (OGG1). In the following year, mammalian (human and other mammals) homologues of OGG1 were identified and cloned [34–40]. In terms of the gene stability associated with DNA damage and its repair systems, the prevalence of 8-oxo-Gua repair enzymes highlights the primary importance of 8-oxo-Gua and OGG1 in most living organisms.

OGG1 removes 8-oxo-Gua from damaged DNA in the base excision repair (BER) process, to prevent the GC-to-TA transversion type of point mutation (Figure 2B) [34,35,41]. The GC-to-TA point mutation is commonly observed in the tumor suppressor *p53* gene in human cancers, such as lung cancer [42–45]. In addition, Hussain and Harris reported that 33% of the somatic *p16^INK4^* mutations in human cancers were GC-to-TA transversions [46]. Moreover, mutations of the *OGG1* gene in human lung cancer have also been reported [36,47–49]. It is reasonable to assume that the *OGG1* mutation may be associated with the GC-to-TA point mutations that occur in the tumor-associated genes of living organisms. OGG1 is known to have glycosylase activity for other lesions, in addition to 8-oxo-Gua. Jensen *et al.* reported that OGG1 also incised 8-oxoadenine opposite cytosine in nuclei and mitochondria [50]. This evidence suggests that OGG1 plays a fundamental role in maintaining DNA stability.

As a line of defense against mutagenesis or carcinogenesis, a repair system, called the GO system, acts in response to 8-oxo-Gua generation [51]. The GO system contains three main enzymes: OGG1, MutY homolog (MUTYH), and MutT homolog 1 (MTH1). MUTYH excises adenine incorporated opposite 8-oxo-Gua, as a mis-match repair enzyme [52]. MTH1 is a clearance enzyme that acts as an oxidized purine nucleoside triphosphatase for 8-oxo-GTP in the nucleotide pool [53]. These three enzymes coordinately prevent 8-oxo-Gua accumulation and, thus, point mutations in DNA. It is noteworthy that the measured level of 8-oxo-Gua depends on the balance between its generation and repair activity. Therefore, to understand 8-oxo-Gua-associated carcinogenesis, studies on both the 8-oxo-Gua levels and its repair ability are required. Indeed, many studies suggested that an imbalance between them might be involved in carcinogenesis.

## 4. Alcohol and 8-Oxoguanine in Liver DNA

**Figure 3:**
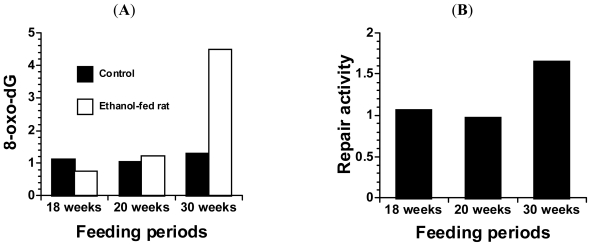
(**A**) 8-Oxo-Gua levels in ethanol-fed rats at 18, 20 and 30 weeks of feeding. The value of 8-oxo-Gua is expressed as 8-oxo-dG/10^5^ dG; (**B**) 8-Oxo-Gua repair activity levels in ethanol-fed rats at 18, 20 and 30 weeks of feeding. The data are expressed as the ratios to the control. The assessment method for 8-oxo-Gua repair activity (endonuclease nicking assay) was described elsewhere [9]. Briefly, the oligonucleotide containing 8-oxo-Gua was used as a substrate for the assay. The total crude extract obtained from animal tissue was incubated with end-labeled double-stranded DNA substrates at 25 °C for 1 hr. After ethanol precipitation, the pellet was dried, dissolved in 10 μL of loading buffer and denatured by heating at 90 °C for 3 min. A 10 μL portion of the sample was applied to a 20% denaturing polyacrylamide gel for electrophoresis. 8-oxo-dG: 7,8-dihydro-8-oxoguanosine.

We analyzed 8-oxo-Gua generation and repair activity in the livers of rats subjected to long-term consumption of ethanol and AD [6]. Male Sprague-Dawley rats (3-week-old) were fed an ethanol beverage whose concentration was increased by 3% every week, starting at 12% and progressing up to 70% (Figure 1). When the concentration reached 50%, the diet of one group was changed from the ND to either AD or ND. At the feeding periods of 18, 20, and 30 weeks (ethanol concentrations were 66, 70, and 70%, respectively), the rats were sacrificed. The livers were immediately excised and the levels of 8-oxo-Gua and its repair activity were measured. The 8-oxo-Gua levels in the livers of ethanol-fed rats at the feeding periods of 18 and 20 weeks showed no change, in comparison to those in the livers of the control rats. The 8-oxo-Gua repair activity also showed no difference at the same feeding periods. However, at the feeding period of 30 weeks, both the 8-oxo-Gua level in the livers of the ethanol-fed rats and its repair activity were increased, as compared to the levels in the control rats (Figure 3). These results indicated that the long-term consumption of a high concentration of alcohol might increase the risks of developing cancer in the liver, derived from oxidative DNA damage.

## 5. Alcohol and 8-Oxoguanine in Esophageal DNA

**Figure 4:**
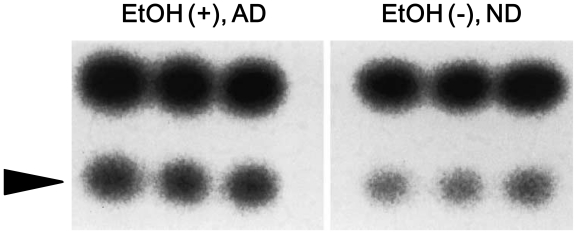
Autoradiogram showing a comparison of the esophageal 8-oxo-Gua repair activities in rats fed ethanol and AD for 30 weeks with those fed ND. Upper band, substrate DNA; lower band (arrow head), excised fragment. The assessment method for the 8-oxo-Gua repair activity (endonuclease nicking assay) was described in the legend of Figure 3. This figure was published in reference [9], Copyright Wiley-Blackwell. ND: normal diet, AD: autoclaved diet.

We analyzed the 8-oxo-Gua generation level and repair activity in the esophagus, in rats subjected to long-term consumption of ethanol and AD [9]. Male Sprague-Dawley rats (3-week-old) were fed an ethanol beverage whose concentration was increased from 12 to 70% over 20 weeks (Figure 1). When the concentration reached 50%, the diet of one group was changed from the ND to AD. At the feeding periods of 20, 25, 30, and 35 weeks, the rats were sacrificed and the 8-oxo-Gua levels and repair activities within the esophagi were measured. After 30 weeks of ethanol- and AD-feeding, significant increases of 8-oxo-Gua and its repair activity were observed in the esophagi, but not in those of the ethanol- and ND-fed rats (Figure 4). This result indicates that the combined effects of long-term ethanol consumption and vitamin deficiency may be involved in inducing oxidative DNA damage in the rat esophagus.

## 6. Chronic Alcohol Consumption Prevents 8-Oxoguanine Accumulation in the Livers of Carcinogen-Treated Mice

**Figure 5:**
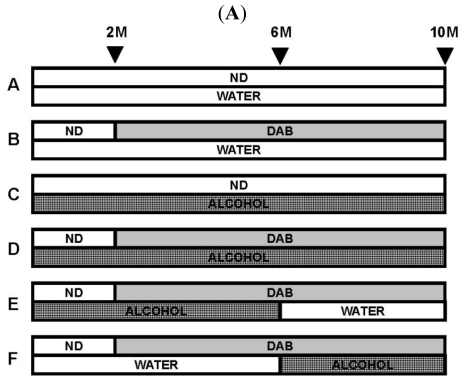

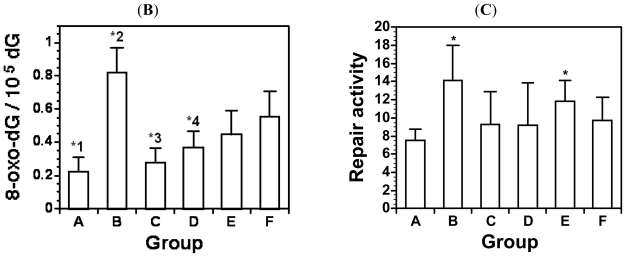
(**A**) The experimental protocol. A: control [ND/water] for 10 months; B: [ND/water] for the first 2 months and [0.06% 3’-MeDAB/water] for the last 8 months; C: [ND/alcohol] for 10 months; D: [ND/alcohol] for the first 2 months and [0.06% 3’-MeDAB/alcohol] for the last 8 months; E: [ND/alcohol] for the first 2 months, [0.06% 3’-MeDAB/alcohol] for the next 4 months, and [0.06% 3’-MeDAB/water] for the last 4 months; F: [ND/water] for the first 2 months, [3’-MeDAB/water] for the next 4 months, and [3’-MeDAB/alcohol] for the last 4 months; (**B**) The levels of 8-oxo-Gua in the DNA of mouse livers. The 8-oxo-Gua value is expressed as the number of 8-oxo-dG per 10^5^ deoxyguanosine. *1: *P* < 0.0005 *vs.* group B, *P* < 0.05 *vs.* group E, *P* < 0.01 *vs.* group F; *2: *P* < 0.0001 *vs.* group C, *P* < 0.005 *vs.* group D, *P* < 0.01 *vs.* group E, *P* < 0.05 *vs.* group F; *3: *P* < 0.05 *vs.* group E, *P* < 0.005 *vs.* group F; *4: *P* < 0.05 *vs.* group F; and (**C**) 8-Oxo-Gua nicking activity in the mouse livers. The activity was calculated as the ratio of the excised fragment intensity to the total substrate (unexcised substrate intensity plus excised fragment intensity). *: *P* < 0.005 *vs.* group A. ND: normal diet, AD: autoclaved diet, 8-oxo-dG: 7, 8-dihydro-8-oxoguanosine. This figure was published in reference [10], Copyright Elsevier.

Recently, we reported the effect of alcohol consumption on 8-oxo-Gua generation in the livers of 3’-methyl-4-dimethylaminoazobenzene (3’-MeDAB)-treated mice [10]. We fed 3’-MeDAB to mice for eight months with/without 12% ethanol, and measured the 8-oxo-Gua generation level and its repair activity in the liver (Figure 5A). 3’-MeDAB is a type of amino azo dye and is highly hepato-carcinogenic [54]. The tumorigenic mechanisms are complex, and are thought to include carcinogen-DNA adduct formation [55,56] and ROS generation [57]. The results showed that 12% ethanol intake attenuated the 8-oxo-Gua accumulation, suggesting that 12% ethanol consumption may reduce the risk of 3’-MeDAB-induced carcinogenesis by decreasing 8-oxo-Gua accumulation (Figures 5B and C).

## 7. Conclusions

As shown in this article, alcohol exhibits at least two different features, in terms of carcinogenesis. One is an enhanced effect for carcinogenesis and the other is an inhibitory effect. Our study indicated that these effects of alcohol consumption on carcinogenesis may depend on the drinking pattern and the diet. To drink alcohol safely, the consumption pattern and the diet should be considered.

## Figures and Tables

**Table 1:** Nutrition value of the diet.

Ingredient	Normal diet	Autoclaved diet
Vitamin B_1_	1.73 mg	0.35 mg (20%)
Vitamin C	19.0 mg	9.0 mg (47%)
Retinol	0.36 mg	0.19 mg (53%)
Vitamin B_12_	4.9 μg	2.6 μg (53%)
Inositol	1.63 mg	109 mg (67%)
Pantoic acid	2.85 mg	2.21 mg (78%)
Vitamin B_6_	1.26 mg	0.99 mg (79%)
Niacin	15.9 mg	14.0 mg (88%)
Vitamin B_2_	1.37 mg	1.21 mg (88%)
Vitamin E	11.0 mg	10.0 mg (91%)
Folic acid	0.15 mg	0.15 mg (100%)
Biotin	47.8 μg	48.1 μg (101%)
Choline	250 mg	260 mg (104%)

% of normal diet. These data were generated in 1984 and provided by Clea Japan, Inc.
